# Abnormal Salivary Total and Oligomeric Alpha-Synuclein in Parkinson’s Disease

**DOI:** 10.1371/journal.pone.0151156

**Published:** 2016-03-24

**Authors:** Giorgio Vivacqua, Anna Latorre, Antonio Suppa, Michela Nardi, Sara Pietracupa, Romina Mancinelli, Giovanni Fabbrini, Carlo Colosimo, Eugenio Gaudio, Alfredo Berardelli

**Affiliations:** 1 Department of Neurology and Psychiatry, Sapienza University, Rome, Italy; 2 Department of Anatomic, Histologic, Forensic and Locomotor Apparatus Sciences, Sapienza University, Rome, Italy; 3 Neuromed Institute, Venafro (IS), Italy; Hertie Institute for Clinical Brain Research and German Center for Neurodegenerative Diseases, GERMANY

## Abstract

In Parkinson’s disease (PD), alpha-synuclein (a-syn) can be detected in biological fluids including saliva. Although previous studies found reduced a-syn total (a-syn_total_) concentration in saliva of PD patients, no studies have previously examined salivary a-syn oligomers (a-syn_olig_) concentrations or assessed the correlation between salivary a-syn_total_, a-syn_olig_ and clinical features in a large cohort of PD patients. Is well known that a-syn_olig_ exerts a crucial neurotoxic effect in PD. We collected salivary samples from 60 PD patients and 40 age- and sex-comparable healthy subjects. PD was diagnosed according to the United Kingdom Brain Bank Criteria. Samples of saliva were analyzed by specific anti-a-syn and anti-oligomeric a-syn ELISA kits. A complete clinical evaluation of each patient was performed using MDS-Unified Parkinson's Disease Rating Scale, Beck Depression Inventory, Montreal Cognitive Assessment and Frontal Assessment Battery. Salivary a-syn_total_ was lower, whereas a-syn_olig_ was higher in PD patients than healthy subjects. The a-syn_olig_/a-syn_total_ ratio was also higher in patients than in healthy subjects. Salivary a-syn_total_ concentration negatively correlated with that of a-syn_olig_ and correlated with several patients’ clinical features. In PD, decreased salivary concentration of a-syn_total_ may reflect the reduction of a-syn monomers (a-syn_mon_), as well as the formation of insoluble intracellular inclusions and soluble oligomers. The combined detection of a-syn_total_ and a-syn_olig_ in the saliva might help the early diagnosis of PD.

## Introduction

Alpha-synuclein (a-syn) is a 140-amino acid protein that is widely expressed in presynaptic terminals of the central nervous system and plays an important role in the pathogenesis of Parkinson’s disease (PD) [1,2]. PD is pathologically characterized by a-syn deposition into neurons and neuronal fibers, which leads to the formation of Lewy Bodies and Lewy Neurities [2,3]. In physiological conditions, a-syn is prevalently expressed as a monomeric form (a-syn_mon_) [4] and is localized in the cytoplasm and in the cellular nuclei or bound to the synaptic vesicles [5,6]. In PD, a-syn_mon_ aggregates into a-syn oligomers (a-syn_olig_) and the oligomers formation, in turn, is followed by oligomers conversion into mature amyloid fibrils, leading to the formation of Lewy bodies and Lewy neurities [7,8]. Soluble a-syn_olig_ are present in larger amounts in brain homogenates of PD patients than in those of healthy subjects [9] and cause neuronal cell death [10], being therefore the main neurotoxic form of a-syn [7,11,12].

Previous studies have investigated a-syn in cerebrospinal fluid (CSF) [13,14,15,16] and in peripheral nerve fibers in biopsies of non-nervous tissues, including gut [17,18,19] and skin [20,21]. A-syn aggregation has also been described in minor salivary glands [22], though no significant difference emerged between PD patients and healthy subjects. By contrast, a-syn aggregation is present in the nervous fibers that innervate the sub-mandibular gland, in PD patients but not in healthy subjects [23].

Saliva is a biological fluid that can easily be collected. Previous studies have investigated a-syn in saliva. Devic and coworkers [24] found that differences in a-syn levels between patients with PD and healthy subjects were not significant, although a trend pointing to lower a-syn levels in PD patients than in healthy subjects was detected. Al-Nimer et al.[25] instead reported that a-syn levels were significantly lower in the saliva of patients with PD than in healthy subjects. Both these studies were performed on small groups of patients with PD and neither investigated a-syn_olig_. Several studies based on CSF have reported that a-syn_olig_ levels are significantly higher in patients with PD than in healthy subjects [9,26,27], which suggests that a-syn_olig_ might be a more reliable diagnostic indicator than total a-syn alone.

The diagnosis of PD is based on clinical criteria [28,29] whose accuracy may be limited, particularly in the early stages of the disease. A validated biomarker would help the clinical diagnosis of PD. Since a-syn_total_ and a-syn_olig_ in peripheral tissues and biological fluids are potential biomarkers of PD, the detection of a-syn in saliva may offer a promising means of molecular diagnosing of PD.

The aim of the present study was to investigate, in a large cohort of PD patients and healthy subjects, a-syn_total_ and a-syn_olig_ levels in saliva to assess whether salivary a-syn can be used to differentiate PD patients from healthy subjects, and whether a-syn_total_ and a-syn_olig_ concentrations correlate with the clinical scores of PD patients. Therefore, we tested a-syn in a group of 60 PD patients and compared the results with those in a group of 40 age- and sex-comparable healthy subjects.

## Methods

### Subjects

After receiving a full explanation of the aims of the study, participants (both patients and healthy subjects) gave their written informed consent. The consent form was previously approved by the Institutional Review Board of the Sapienza University of Rome. The study was approved by the Ethical Commmitte of the Sapienza University of Rome and all clinical investigations are conformed to the Declaration of Helsinki.

Sixty patients with PD (31 males, 29 females; mean age ±SD: 66.3±8.78, range 53–82 years; mean±SD age at onset: 60.4±9.2, range 28–77 years) were recruited at the Movement Disorders outpatient clinic of the Department of Neurology and Psychiatry, Sapienza University of Rome, Italy. Forty healthy subjects, who were age- and sex-comparable with the PD patients (22 males, 18 females; mean age±SD: 68.3±7.9, range 51–83 years), were also enrolled in the study. We designed to enroll a total number of 60 patients with PD and 40 healthy subjects on the basis of a preliminary statistical power analysis (sample size estimation) that demonstrated a minimum number of at least 40 participants to reach statistical significant differences when comparing the two groups. Diagnosis of PD was based on the Queen Square Brain Bank Criteria [28,29]. PD diagnosis was also confirmed with follow-up clinical evaluations [29]. Demographic and clinical information was collected through a face-to-face questionnaire (S1 Table), and a complete neurological examination was performed. The data collected for each patient included: stage of the disease, scored with the Hoehn & Yahr scale (HY); severity of disease, assessed with the MDS-Unified Parkinson’s Disease Rating Scale (MDS-UPDRS) (part 1–4); mood, assessed with the Beck Depression Inventory (BDI-II); cognitive impairment, assessed with the Montreal Cognitive Assessment (MOCA) and the Frontal Assessment Battery (FAB). Current pharmacological treatment for PD was also assessed and calculated as L-Dopa equivalent daily dose (LEDD), for each drug, as previously reported [30]. We calculated LEDDs in order to avoid methodological biases due to the large variability in drug subclasses taken by the different patients. Patients were clinically evaluated while they were under their usual medical treatment and were in their ON state. Patients affected by atypical or secondary parkinsonisms were excluded from the study. All patients enrolled in the study had a MOCA score higher than 18 and a FAB score higher than 12 [31,32]. Participants (whether patients or healthy subjects) affected by cardiovascular and cerebrovascular diseases, diabetes mellitus, autoimmune diseases, chronic inflammatory diseases, hematological neoplasms and solid tumors were also excluded from this study, as well subjects affected by salivary gland and oral cavity pathologies.

### Sample collection

We collected a minimum quantity of 3 ml of saliva from each PD patient and healthy subject. At the time of collection of the saliva sample, subjects had fasted for 60 minutes, had not smoked in the preceding 4 hours and had not drunk alcohol in the previous 12 hours. Samples with blood contamination were excluded from the study. Saliva was collected by instructing the patients to drool into a 50 ml vial, which was immediately placed on ice in order to block proteolytic activity.

Samples were then placed in 10 ml falcon-type tubes and centrifuged for 15 min at 2,600 x g at 4°C to obtain a first clarification, and then for another 15 min at 15,000 x g at 4°C to remove residual particles, such as bacteria and desquaming mucosal cells. After centrifugation, the supernatant was transferred into new 10 ml falcon-type tubes and each sample was treated with a protease activity-inhibiting buffer (Sigma Aldrich, St.Luis, MO, USA, Cat #P2714), at a concentration of 100 μL for 1 ml of saliva. Each sample was then transferred into a 1 ml Heppendorf-type test tube, thus providing six 300 μl-samples from each patient. Samples were stored at -80° before the ELISA investigation. The samples were collected according to the protocol adopted by Devic and coworkers [24] in previous works on saliva.

To verify possible changes in salivary a-syn concentration (a-syn_total_ and a-syn_olig_) in independent samples, we also collected 2 independent sample sets in the same day (8 a.m. and 4 p.m.) in a subgroup of 10 healthy subjects and 10 patients with PD. Finally, to verify the consistency of our analytical results, we have also measured a-syn concentration two times in the same salivary sample in the same subgroup of 10 healthy subjects and 10 patients with PD.

### ELISA analysis of samples

Samples were analyzed at the laboratory of Immunohistochemistry of the Department of Human Anatomy, Sapienza University of Rome.

A-syn_total_ and a-syn_olig_ were assessed in saliva by enzyme immunoassay (ELISA). The concentration of the protein was determined by spectrometric measurement at 450 nm in an appropriate microplate reader. We used this method to draw a standard curve based on the optical density resulting from the presence of a-syn_total_ and of a-syn_olig_ in the samples, and we calculated the concentration of the two protein variants in each sample. Each standard curve of optical density corresponds to each optical density value of a specific value in the pg or ng of the protein concentration. This method allowed us to achieve a range of linearity based on the concentration of a-syn_total_ and a-syn_olig_, and a range of concentration values that could be used to differentiate patients with PD from healthy subjects.

We used the anti-alpha-Synuclein Quantitative ELISA Kit (SensoLyte 55550) to determine a-syn_total_. The ELISA kit has been already used to determine a-syn_total_ in saliva in a previous study in patients with PD [25]. To determine a-syn_olig_ we used the Human a-syn oligomer ELISA Kit (MyBioSource, MBS730762). This ELISA kit is specific for detecting a-syn_olig_, with a very low cross-reactivity for a-syn_mon_ (for detailed information: data in S1 Text, by courtesy of MyBioSource lab. Inc., San Diego, CA).

Each sample from PD patient and healthy subject has been analyzed in three different wells and the results are the averaged a-syn_total_ and a-syn_olig_ concentration from the three different wells.

During ELISA analysis, we cautiously used all the methodological precautions to minimize “*in vitro”* aggregation or disaggregation of a-syn_olig_, including stable physical conditions during all experimental procedures (pH 7.44, temperature 37°C, 0,9% electrolyte concentrations of solutions).

### Data analysis and atatistics

Each optical density value, obtained from the ELISA test, was normalized with the optical density of blank controls. Concentrations of a-syn_total_ and a-syn_olig_ were calculated following the linearity curve obtained in the ELISA test. The a-syn_olig_/a-syn_total_ ratio was calculated in each patient and healthy subject.

Mann-Whitney U test was used to compare the salivary concentration of a-syn_total_ (pg/ml), a-syn_olig_ (ng/ml) and the a-syn_total_/a-syn_olig_ ratio in patients with PD and healthy subjects. Data are presented in linear bar diagrams. Mann-Whitney U test was also used to compare the salivary concentration of a-syn_total_ and a-syn_olig_ in the 2 independent sample sets, collected in the same day (8 a.m. and 4 p.m.), in the subgroup of 10 healthy subjects and 10 PD patients. Finally, Mann-Whitney U test was again used to compare the salivary concentration of a-syn_total_ and a-syn_olig_, measured in two independent experiments on the same salivary sample, in the same subgroup of 10 healthy subjects and 10 PD patients.

The Spearman's Rank Correlation Coefficient was used to detect any correlations between the PD patients’ clinical data, including age, disease duration, H&Y, MDS-UPDRS (part 1–4), BDI-II, MOCA, FAB and LEDD, and the biochemical analysis. Holm’s correction for multiple comparisons was used to discover false significance. P values <0.05 were considered to indicate statistical significance.

## Results

The cohort of patients with PD we studied had a mean disease duration of 6.7±10.4 years, an H&Y score of 1.8±0.75. The MDS-UPDRS score was 9.18±5.95 (part 1); 9.93±9.8 (part 2); 23.7±15.48 (part 3) and 2.2±4.3 (part 4). BDI-II score was 10.55±7.54, MOCA score was 26.08±2.59, FAB score was 16.91±1.44 and finally, LEDD was 460±179.81 mg/day (S1 Table).

**Fig 1:**
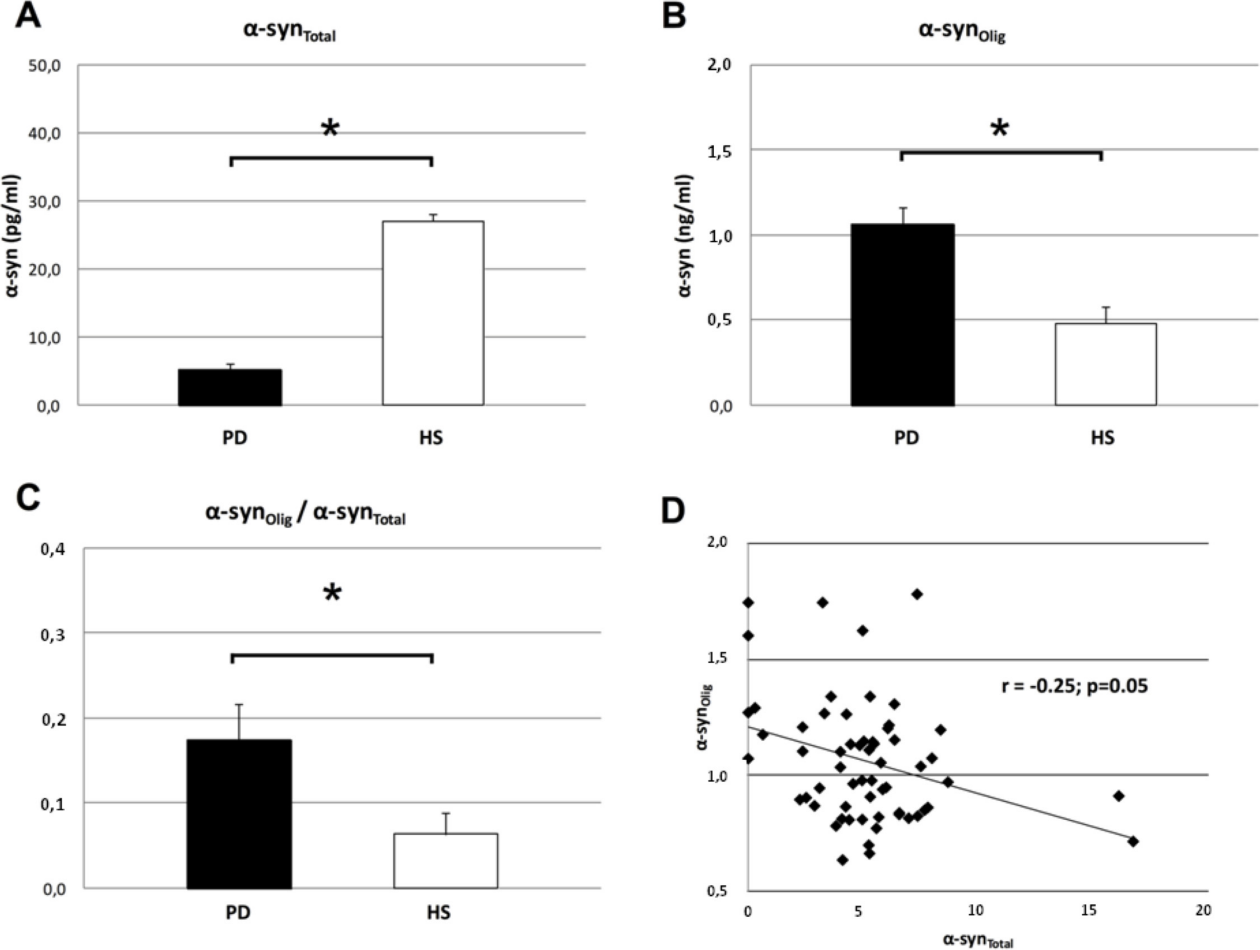
Salivary a-synuclein in PD patients and healthy subjects. Salivary a-syn in patients with Parkinson’s disease (PD) and healthy subjects (HS). Each histogram corresponds to the mean concentration of α-syn_total_ (panel A), α-syn_olig_ (panel B) and the α-syn_total_/α-syn_olig_ ratio (panel C) in the saliva of patients with PD (black histogram) and HS (white histogram). Vertical bars denote standard deviation. Asterisk denote significant differences. Scatter-plot (panel D) shows the negative correlation between α-syn_total_ and α-syn_olig_ in patients with PD.

The Mann-Whitney U test revealed significant lower α-syn_total_ levels in the saliva of patients with PD than in that of healthy subjects (z = -7.98; p<0.01, Fig 1A). A-syn_total_ levels in the saliva were 5.08±3.01ρg/ml in patients with PD and 31.3±22.4 ρg/ml in healthy subjects. By contrast, the Mann-Whitney U test showed that α-syn_olig_ was significantly higher in the saliva of patients with PD than in that of healthy subjects (z = -7.82; p<0.01, Fig 1B). The average α-syn_olig_ concentration was 1.062±0.266 ng/ml in patients with PD and 0.498±0.203 ng/ml in healthy subjects. The Mann-Whitney U test also revealed comparable salivary concentration of a-syn_total_ and a-syn_olig_ in the 2 independent sample sets, collected in the same day (8 a.m. and 4 p.m.), in the subgroup of healthy subjects and PD patients (all p values < 0.05). Finally, the Mann-Whitney U test again revealed comparable salivary concentration of a-syn_total_ and a-syn_olig_, measured in two independent experiments on the same salivary sample, in the same subgroup of 10 healthy subjects and 10 PD patients (all p values < 0.05).

The α-syn_olig_/α-syn_total_ ratio was significantly higher in patients with PD than in healthy subjects (z = -8.30; p<0.01, Fig 1C). The mean ratio in patients with PD was 0.174±0.044, whereas the mean ratio in healthy subjects was 0.065±0.027.

**Fig 2:**
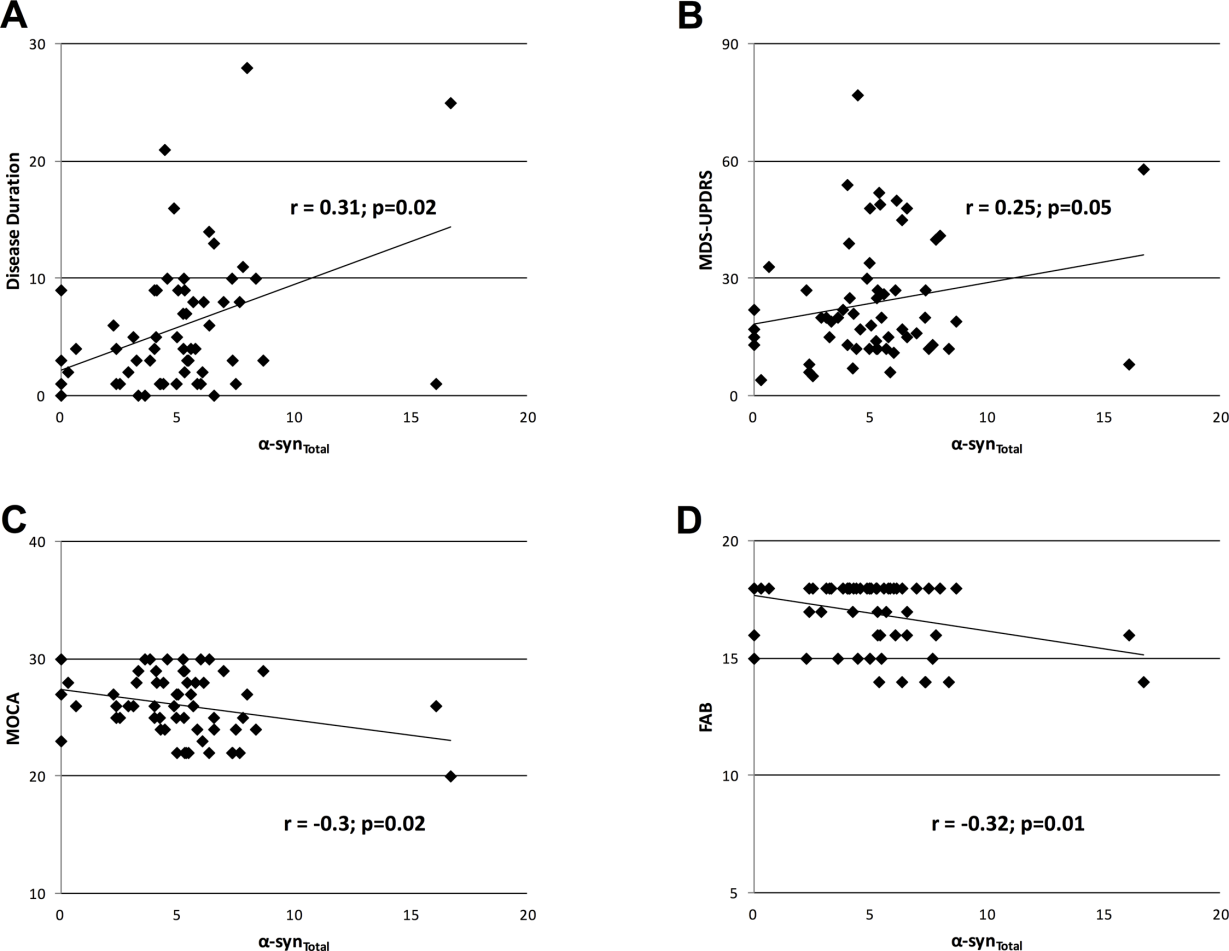
Correlations of total a-syn with PD patients’ clinical features. Scatter-plots showing the correlation between α-syn_total_ and patients’ disease duration (DD) (panel A), Movement Disorder Society-Unified Parkinson’s Disease Rating Scale (MDS-UPDRS) (part 1–4) (panel B), Montreal Cognitive Assessment (MOCA) (panel C) and Frontal Assessment Battery (FAB) scores (panel D) in patients with PD.

The Spearman's Rank Correlation Coefficient revealed a negative correlation between α-syn_total_ and α-syn_olig_ (r = -0.25; p = 0.05; Fig 1D), a positive correlation between α-syn_total_ and disease duration (r = 0.31; p = 0.02; Fig 2A), H&Y (r = 0.29; p = 0.02) and MDS-UPDRS total score (part 1–4) (r = 0.25; p = 0.05; Fig 2B). Spearman's Rank Correlation Coefficient revealed a trend for a positive correlation between α-syn_total_ and MDS-UPDRS part 2 (r = 0.23; p = 0.07) and part 4 scores (r = 0.24; p = 0.06), whereas it failed to detect any significant correlation with MDS-UPDRS part 1 (r = 0.15; p = 0.26) and part 3 scores (r = 0.20; p = 0.11). Spearman's Rank Correlation Coefficient also found a positive correlation between α-syn_total_ and LEDD (r = 0.4; p<0.01), and a negative correlation, between α-syn_total_ and MOCA (r = -0.3; p = 0.02; Fig 2C) and FAB scores (r = -0.32; p = 0.01; Fig 2D). Finally, the Spearman's Rank Correlation Coefficient did not detect any correlation between α-syn_olig_ or the α-syn_olig_/α-syn_total_ ratio and the PD patients’ clinical data.

## Discussion

In the present study, we found that salivary a-syn_total_ is significantly lower in patients with PD than in healthy subjects. Conversely, salivary a-syn_olig_ levels are higher in patients with PD than in healthy subjects. Accordingly, the a-syn_olig_/a-syn_total_ ratio is significantly higher in patients with PD than in healthy subjects. We also found a negative correlation between salivary a-syn_total_ and a-syn_olig_, as well as correlation between a-syn_total_ and several clinical features of the patients studied.

We made every effort to ensure that the patients with PD had varying degrees of disease severity (H&Y stage I to III) and disease duration (range 1 to 28 years). Any PD patient or healthy subject affected by other systemic diseases such as diabetes, cardiovascular diseases, autoimmune diseases or chronic inflammatory diseases, in whom oxidative stress and a-syn misfolding may occur, were excluded. Blood contamination of the samples, which is known to affect the significance of a-syn detection [33], was carefully avoided. Moreover, we used two commercial ELISA kits, one designed for a-syn and already used to detect a-syn_total_ in saliva [25] and another, specifically designed to detect a-syn_olig_ (see data in S1 Text) in serum and biological fluids.

The first finding in this study, on a large cohort of PD patients and healthy subjects, is the reduction in a-syn_total_ in the saliva of patients with PD compared with healthy subjects. This finding is fully in agreement with two previous observations in relatively small cohorts of patients with PD [24,25]. It is important to note that determination of a-syn_total_ through ELISA or Immuno-essay, as performed in this paper and in previous studies [24,25], likely underestimate the total a-syn concentration in the saliva. Since the antibodies generally used to detect a-syn_total_ are aimed at linear epitopes on the molecule of a-syn, antibodies may only detect the unaggregated forms of the protein. By contrast, these antibodies may fail to detect aggregated forms of a-syn, such as a-syn_olig_, owing to the masking of epitopes by changes in the three-dimensional conformation of the protein, leading to a-syn aggregation. In the present study, we therefore considered the a-syn_total_ concentration largely as an estimate of the a-syn_mon_, and we used a different ELISA kit, specifically designed for oligomers of a-syn,to detect a-syn_olig_,.

The reduction in salivary a-syn_total_, and therefore in a-syn_mon_, in patients with PD compared with healthy subjects may be explained by considering a-syn aggregation. Neuropathological studies have shown that in the early stages of PD intracellular aggregation of a-syn occurs in several brainstem nuclei, including the solitary tract nucleus, the dorsal motor nucleus of vagus [3,34] and probably the superior and inferior salivary nuclei and the parasympathetic salivary ganglia [34]. It suggests that a-syn may spread from neuronal cell bodies of salivary neurons, along axons, to the synaptic terminals around the epithelial cells of salivary glands, where it also accumulates in the saliva [24,25]. It is therefore possible that the reduced a-syn_total_ concentration detected in the saliva of patients with PD is due to an intracellular and axonal aggregation of a-syn in the neurons of the salivary nuclei or salivary ganglia.

A novel finding in this study is that, unlike a-syn_total_ and therefore a-syn_mon_, the concentration of salivary a-syn_olig_ is higher in PD patients than in healthy subjects. Accordingly, the a-syn_olig_/a-syn_total_ ratio was also higher in PD patients than in healthy subjects. Pathogenetic mechanisms may explain the increased concentration of syn_olig_ observed in the saliva of PD patients. Indeed, in physiological conditions, there is equilibrium between monomers and oligomers of a-syn that reflects the efficacy of the cellular cleaning mechanisms from aggregated and misfolded proteins [35,36]. In patients with PD, there is an altered turnover of a-syn [37], leading to the accumulation of a-syn_olig_ at both the intracellular and extracellular levels. Moreover, we found that the concentration of salivary a-syn_total_ negatively correlates with that of a-syn_olig_, which further supports the hypothesis that a-syn_mon_ are reduced in the saliva of PD patients because a-syn_mon_ are consumed during the formation of a-syn aggregates, including a-syn_olig_. A-syn_olig_ are multimeric, aggregated forms of a-syn, with an higher molecular weight than a-syn_mon_ (unaggregated forms of a-syn), explaining the salivary concentration in ng rather than in pg as reported for a-syn_total_, which may estimate, in turn, essentially a-syn_mon_.

In patients with PD, we found a positive correlation between a-syn_total_ and disease duration, H&Y, MDS-UPDRS (part 1–4) total score and LEDDs, and a negative correlation between a-syn_total_ and the MOCA and FAB scores. A possible explanation for these findings is that, as the disease progresses, increased synaptic degeneration leads to an increased extracellular release of a-syn into the interstitial fluids, including saliva. Our results do not support the trend of negative correlation between a-syn_total_ concentration in saliva and MDS-UPDRS previously reported by Devic and coworkers [24]. The reason for these variable results may reflect methodological differences including the different number of patients studied. Furthermore, the observation that salivary a-syn_total_ concentration is lower in patients with a lower H&Y and MDS-UPDRS scores (early stages of the disease) than in patients with higher H&Y and MDS-UPDRS scores (late stages of the disease) suggest that the evaluation of salivary a-syn_total_ concentration might be an helpful tool in the diagnosis of PD particularly in the early stage of the disease. Despite a-syn_total_, we failed to detect a correlation between a-syn_olig_ in saliva and the clinical features of the PD patients. This finding is difficult to explain. A possible explanation could be the well-known molecular heterogeneity of a-syn_olig_ [12,37]. This could lead to a widespread variability of a-syn_olig_ conformation in the saliva of PD patients at different stages of the disease. In line with this hypothesis, further studies of molecular biology may be needed to detect the different “subclasses” of a-syn_olig_ in saliva and the correlation of each of them with clinical features of PD patients.

The data on saliva that emerge from our study are in keeping with those reported in previous studies based on CSF [13,14,15,16]. Moreover, the correlations between salivary a-syn_total_ and clinical severity scores are supported by recent reports in CSF, in which higher levels of α-syn_total_ correlate with a worsening in both motor symptoms [38] and cognitive performances [39]. Besides the previous studies on a-syn_total_ our finding of increased a-syn_olig_ levels in the saliva of PD patients is fully in line with the results of previous papers [26,27,40], who reported that a-syn_olig_ levels are significantly higher in the CSF of PD patients than in that of healthy subjects. Our results are also in keeping with recent studies showing increased concentration of pathological aggregated forms of a-syn in the CSF of PD patients [41], using a new monoclonal antibody (5G4 a-syn) specific for aggregated forms of a-syn. Conversely, by using the same 5G4 a-syn antibody in the serum of PD patients, Maetzler et al. (2014) [42] found comparable concentration of 5G4 a-syn and a-syn_total_. Since CSF and saliva, but not serum, are fluids in contact with neuronal fibers and therefore likely affected by a-syn secretion, the serum concentration of different forms of a-syn is difficult to compare with those measured in CSF and saliva. The results obtained from CSF and saliva may confirm that the oligomeric form of a-syn is the prevalent a-syn species in the extracellular fluids of PD patients and that a-syn_total_ levels are reduced following the formation of a-syn aggregates and the consequent reduction in a-syn_mon_.

Our study has certain important limitations. We found that a-syn_total_ and a-syn_olig_ concentrations overlap in some healthy subjects and PD patients. Moreover, some biochemical limitations may affect the ELISA analysis of salivary samples. First, saliva is a biological fluid that contains proteolytic and glycolytic enzymes, possibly influencing the concentration of a-syn in saliva through proteolitic digestion. Although we used a specific protease inhibitor to prevent enzymatic digestion of proteins and intermolecular bonds, some residual enzymatic activity may be still present. Second, a-syn_olig_ are heterogeneous and unstable molecules that are altered by dissociation and conformational changes [37], which may therefore markedly, and unpredictably, affect antigen-antibody binding. Therefore, a misleading by antibodies used and a cross-reaction with a-syn_mon_ cannot be excluded, despite the specificity of the ELISA kit used for the detection of a-syn_olig_. In addition, it is known that a-syn_olig_ may be differentiated in several subclasses and the different aggregation mechanisms of a-syn lead to the lipophilic nature of specific subclasses of a-syn_olig_ [7,12]. It is difficult however, to measure lipophilic aggregates of a-syn such as large beta-sheet aggregates of a-syn in solutions. The lack of detection of lipophilic a-syn_olig_ is a limitation of the study. Finally, although the ELISA kits we used were not specifically validated for saliva, it is important to note that these ELISA kits have been validated in serum and biological fluids, as reported in the data sheet of the products. Furthermore, when testing saliva, we used all precautions to avoid methodological drawbacks due to the specific biophysical and biochemical characteristics of saliva including application of proteolytic inhibitor to prevent the action of proteolytic enzymes of saliva on the proteins.

Previous studies have demonstrated that, in the early stage of PD, patients may manifest hyposialorrhea, whereas in more advanced stage of the disease, patients may manifest hypersialorrhea [43,44]. Accordingly, in patients with PD, abnormal salivary secretion in terms of volume and composition [43,44] might influence the salivary concentration of a-syn_total_ and a-syn_olig_. This hypothesis seems, however, unlikely because in our study we found a lack of correlation between a-syn_total_ and a-syn_olig_ and sialorrhea as scored by the MDS-UPDRS item 2.2. This finding suggests that concentration of a-syn in saliva is not related to salivary secretion rate. However, future investigations correlating objective salivary secretion scores [43] and salivary a-syn_total_ and a-syn_olig_ concentration will help to clarify whether salivary a-syn_total_ and a-syn_olig_ also reflect the salivary secretion rate in PD.

In conclusion, although our study was performed by ELISA kits not specifically validated in saliva, our results suggest that salivary a-syn_total_ is reduced in patients affected by PD and provides the first evidence that both a-syn_olig_ levels and the a-syn_olig_/a-syn_total_ ratio are increased in the saliva of PD patients. Considering the heterogeneity of a-syn_olig_, comparative studies with different anti-oligomeric a-syn specific antibodies are needed to definitively confirm our data. Further studies are required to confirm whether a-syn in saliva may be used as a biomarker for PD [45] and whether it correlates with disease progression and severity. Future studies will clarify whether the combined analysis of a-syn_total_ and a-syn_olig_ in the saliva might help the early diagnosis of PD and the differential diagnosis between PD and atypical parkinsonisms including Multiple System Atrophy (MSA) and Progressive Supranuclear Palsy (PSP).

## Supporting Information

S1 Table Clinical data of PD patients enrolled in the study. (DOC)

S1 Text Detailed informations about anti a-syn oligomers ELISA kit (MyBioSource, MBS730762). (PDF)
