# Supplementary material for: Abnormal Salivary Total and Oligomeric Alpha-Synuclein in Parkinson’s Disease
Source: PLoS One. 2016 Mar 24;11(3):e0151156. doi: 10.1371/journal.pone.0151156 (PMC4807094; doi:10.1371/journal.pone.0151156)
Supplement: S1 Table — (DOC) [file pone.0151156.s001.doc]

**S1 Table**

Demographic and clinical features of patients with Parkinson’s disease (PD). DD: disease duration; HY: Hoehn & Yahr scale; MDS-UPDRS (part 1-4): Movement Disorder Society-Unified Parkinson’s Disease Rating Scale. BDI-II: Beck Depression Inventory; MOCA: Montreal Cognitive Assessment scale; FAB: Frontal Assessment Battery; LEDD: L-Dopa equivalent daily dose.

|  |  | ***Age*** | ***DD*** | ***HY*** | ***MDS-UPDRS***  ***(part 1)*** | ***MDS-UPDRS***  ***(part 2)*** | ***MDS-UPDRS***  ***(part 3)*** | ***MDS-UPDRS***  ***(part 4)*** | ***BDI-II*** | ***MOCA*** | ***FAB*** |
| --- | --- | --- | --- | --- | --- | --- | --- | --- | --- | --- | --- |
| ***1*** | ***F*** | ***55*** | ***8*** | ***3*** | ***17*** | ***20*** | ***50*** | ***8*** | ***13*** | ***28*** | ***18*** |
| ***2*** | ***M*** | ***68*** | ***3*** | ***2*** | ***4*** | ***5*** | ***20*** | ***0*** | ***8*** | ***22*** | ***15*** |
| ***3*** | ***M*** | ***61*** | ***1*** | ***1*** | ***9*** | ***2*** | ***12*** | ***0*** | ***15*** | ***24*** | ***18*** |
| ***4*** | ***F*** | ***76*** | ***11*** | ***2,5*** | ***10*** | ***17*** | ***40*** | ***17*** | ***6*** | ***25*** | ***16*** |
| ***5*** | ***M*** | ***65*** | ***2*** | ***1*** | ***4*** | ***1*** | ***12*** | ***0*** | ***10*** | ***29*** | ***17*** |
| ***6*** | ***F*** | ***65*** | ***2*** | ***2*** | ***9*** | ***2*** | ***4*** | ***0*** | ***18*** | ***28*** | ***18*** |
| ***7*** | ***F*** | ***80*** | ***1*** | ***2*** | ***2*** | ***2*** | ***13*** | ***0*** | ***8*** | ***27*** | ***18*** |
| ***8*** | ***M*** | ***72*** | ***1*** | ***1*** | ***2*** | ***0*** | ***5*** | ***0*** | ***5*** | ***25*** | ***18*** |
| ***9*** | ***M*** | ***78*** | ***1*** | ***1*** | ***3*** | ***3*** | ***34*** | ***0*** | ***10*** | ***27*** | ***18*** |
| ***10*** | ***M*** | ***67*** | ***4*** | ***2*** | ***23*** | ***23*** | ***48*** | ***9*** | ***24*** | ***22*** | ***15*** |
| ***11*** | ***F*** | ***47*** | ***0*** | ***1*** | ***5*** | ***2*** | ***19*** | ***0*** | ***2*** | ***29*** | ***18*** |
| ***12*** | ***F*** | ***69*** | ***10*** | ***2*** | ***8*** | ***9*** | ***12*** | ***7*** | ***4*** | ***24*** | ***14*** |
| ***13*** | ***M*** | ***82*** | ***9*** | ***1,5*** | ***9*** | ***7*** | ***22*** | ***0*** | ***5*** | ***23*** | ***15*** |
| ***14*** | ***F*** | ***67*** | ***1*** | ***1*** | ***4*** | ***0*** | ***6*** | ***0*** | ***5*** | ***26*** | ***18*** |
| ***15*** | ***F*** | ***75*** | ***3*** | ***2*** | ***1*** | ***1*** | ***19*** | ***0*** | ***10*** | ***29*** | ***18*** |
| ***16*** | ***F*** | ***66*** | ***0*** | ***1,5*** | ***5*** | ***1*** | ***20*** | ***0*** | ***11*** | ***30*** | ***15*** |
| ***17*** | ***M*** | ***58*** | ***4*** | ***2*** | ***18*** | ***19*** | ***33*** | ***5*** | ***20*** | ***26*** | ***18*** |
| ***18*** | ***F*** | ***54*** | ***3*** | ***2*** | ***5*** | ***7*** | ***49*** | ***0*** | ***8*** | ***28*** | ***16*** |
| ***19*** | ***F*** | ***70*** | ***14*** | ***3*** | ***27*** | ***27*** | ***45*** | ***2*** | ***30*** | ***22*** | ***14*** |
| ***20*** | ***M*** | ***53*** | ***8*** | ***1,5*** | ***10*** | ***13*** | ***16*** | ***9*** | ***9*** | ***29*** | ***18*** |
|  |  | ***Age*** | ***DD*** | ***HY*** | ***MDS-UPDRS***  ***(part 1)*** | ***MDS-UPDRS***  ***(part 2)*** | ***MDS-UPDRS***  ***(part 3)*** | ***MDS-UPDRS***  ***(part 4)*** | ***BDI-II*** | ***MOCA*** | ***FAB*** |
| ***21*** | ***M*** | ***68*** | ***4*** | ***1*** | ***6*** | ***1*** | ***15*** | ***0*** | ***8*** | ***28*** | ***18*** |
| ***22*** | ***F*** | ***66*** | ***3*** | ***1*** | ***8*** | ***5*** | ***15*** | ***0*** | ***13*** | ***28*** | ***18*** |
| ***23*** | ***M*** | ***56*** | ***28*** | ***4*** | ***20*** | ***34*** | ***41*** | ***14*** | ***28*** | ***27*** | ***18*** |
| ***24*** | ***F*** | ***86*** | ***2*** | ***2*** | ***11*** | ***15*** | ***27*** | ***0*** | ***10*** | ***23*** | ***16*** |
| ***25*** | ***F*** | ***68*** | ***4*** | ***2*** | ***2*** | ***1*** | ***8*** | ***0*** | ***4*** | ***25*** | ***17*** |
| ***26*** | ***M*** | ***77*** | ***10*** | ***3*** | ***9*** | ***16*** | ***20*** | ***0*** | ***15*** | ***22*** | ***14*** |
| ***27*** | ***F*** | ***72*** | ***16*** | ***3*** | ***17*** | ***20*** | ***30*** | ***0*** | ***6*** | ***26*** | ***18*** |
| ***28*** | ***M*** | ***76*** | ***1*** | ***2*** | ***12*** | ***9*** | ***21*** | ***0*** | ***19*** | ***24*** | ***18*** |
| ***29*** | ***F*** | ***56*** | ***0*** | ***1*** | ***8*** | ***3*** | ***15*** | ***0*** | ***14*** | ***27*** | ***18*** |
| ***30*** | ***M*** | ***76*** | ***9*** | ***2*** | ***13*** | ***17*** | ***54*** | ***6*** | ***8*** | ***25*** | ***18*** |
| ***31*** | ***M*** | ***63*** | ***3*** | ***1*** | ***11*** | ***9*** | ***17*** | ***0*** | ***7*** | ***30*** | ***16*** |
| ***32*** | ***F*** | ***59*** | ***5*** | ***1*** | ***12*** | ***5*** | ***20*** | ***0*** | ***13*** | ***26*** | ***18*** |
| ***33*** | ***F*** | ***50*** | ***4*** | ***1*** | ***4*** | ***7*** | ***13*** | ***0*** | ***3*** | ***26*** | ***18*** |
| ***34*** | ***F*** | ***57*** | ***1*** | ***1*** | ***3*** | ***2*** | ***8*** | ***0*** | ***3*** | ***26*** | ***18*** |
| ***35*** | ***M*** | ***69*** | ***21*** | ***4*** | ***24*** | ***43*** | ***77*** | ***15*** | ***11*** | ***24*** | ***15*** |
| ***36*** | ***F*** | ***62*** | ***1*** | ***1*** | ***7*** | ***1*** | ***12*** | ***0*** | ***42*** | ***28*** | ***18*** |
| ***37*** | ***F*** | ***58*** | ***0*** | ***2*** | ***9*** | ***6*** | ***15*** | ***0*** | ***14*** | ***24*** | ***17*** |
| ***38*** | ***M*** | ***81*** | ***13*** | ***3*** | ***9*** | ***28*** | ***48*** | ***0*** | ***8*** | ***25*** | ***16*** |
| ***39*** | ***M*** | ***72*** | ***2*** | ***2,5*** | ***10*** | ***7*** | ***20*** | ***0*** | ***15*** | ***26*** | ***17*** |
| ***40*** | ***M*** | ***68*** | ***1*** | ***2*** | ***1*** | ***3*** | ***12*** | ***0*** | ***7*** | ***25*** | ***18*** |
| ***41*** | ***M*** | ***64*** | ***1*** | ***1*** | ***7*** | ***0*** | ***7*** | ***0*** | ***7*** | ***25*** | ***17*** |
| ***42*** | ***F*** | ***65*** | ***4*** | ***2*** | ***11*** | ***7*** | ***26*** | ***3*** | ***4*** | ***27*** | ***18*** |
| ***43*** | ***F*** | ***64*** | ***10*** | ***2*** | ***3*** | ***1*** | ***25*** | ***0*** | ***4*** | ***25*** | ***18*** |
| ***44*** | ***M*** | ***54*** | ***9*** | ***1,5*** | ***5*** | ***9*** | ***18*** | ***0*** | ***2*** | ***27*** | ***18*** |
| ***45*** | ***F*** | ***77*** | ***9*** | ***2*** | ***17*** | ***17*** | ***27*** | ***0*** | ***12*** | ***22*** | ***16*** |
| ***46*** | ***F*** | ***65*** | ***4*** | ***1*** | ***2*** | ***10*** | ***12*** | ***0*** | ***0*** | ***29*** | ***18*** |
| ***47*** | ***F*** | ***64*** | ***3*** | ***1,5*** | ***9*** | ***4*** | ***22*** | ***0*** | ***8*** | ***30*** | ***18*** |
| ***48*** | ***M*** | ***72*** | ***3*** | ***1,5*** | ***8*** | ***5*** | ***27*** | ***0*** | ***8*** | ***22*** | ***14*** |
| ***49*** | ***M*** | ***62*** | ***7*** | ***1*** | ***5*** | ***14*** | ***14*** | ***0*** | ***18*** | ***30*** | ***18*** |
| ***50*** | ***M*** | ***53*** | ***5*** | ***2,5*** | ***11*** | ***14*** | ***39*** | ***12*** | ***12*** | ***29*** | ***18*** |
| ***51*** | ***F*** | ***66*** | ***8*** | ***2*** | ***11*** | ***4*** | ***13*** | ***0*** | ***16*** | ***22*** | ***15*** |
| ***52*** | ***M*** | ***65*** | ***8*** | ***2*** | ***9*** | ***27*** | ***12*** | ***8*** | ***8*** | ***26*** | ***17*** |
| ***53*** | ***F*** | ***54*** | ***6*** | ***1,5*** | ***9*** | ***12*** | ***17*** | ***0*** | ***5*** | ***30*** | ***18*** |
|  |  | ***Age*** | ***DD*** | ***HY*** | ***MDS-UPDRS***  ***(part 1)*** | ***MDS-UPDRS***  ***(part 2)*** | ***MDS-UPDRS***  ***(part 3)*** | ***MDS-UPDRS***  ***(part 4)*** | ***BDI-II*** | ***MOCA*** | ***FAB*** |
| ***54*** | ***M*** | ***60*** | ***1*** | ***1*** | ***9*** | ***1*** | ***11*** | ***0*** | ***9*** | ***30*** | ***18*** |
| ***55*** | ***M*** | ***75*** | ***75*** | ***3*** | ***23*** | ***26*** | ***58*** | ***9*** | ***23*** | ***22*** | ***14*** |
| ***56*** | ***F*** | ***71*** | ***6*** | ***2*** | ***12*** | ***4*** | ***27*** | ***3*** | ***6*** | ***27*** | ***15*** |
| ***57*** | ***F*** | ***64*** | ***10*** | ***2*** | ***4*** | ***5*** | ***17*** | ***0*** | ***4*** | ***30*** | ***18*** |
| ***58*** | ***M*** | ***83*** | ***7*** | ***2*** | ***11*** | ***29*** | ***52*** | ***0*** | ***6*** | ***22*** | ***14*** |
| ***59*** | ***F*** | ***68*** | ***1*** | ***1*** | ***4*** | ***2*** | ***6*** | ***0*** | ***5*** | ***24*** | ***18*** |
| ***60*** | ***M*** | ***62*** | ***9*** | ***2*** | ***10*** | ***12*** | ***25*** | ***5*** | ***7*** | ***28*** | ***18*** |
| ***AV±SD*** |  | ***66.3±8.77*** | ***6.7±10.4*** | ***1.8±0.75*** | ***9,18±5.95*** | ***9,93±9.80*** | ***23.7±15.48*** | ***2,20±4.30*** | ***10,55±7.54*** | ***26,08±2.59*** | ***16,95±1.44*** |

**Supplemental Material 2 (attached PDF file):** Additional informations about the alpha-Synuclein oligomer ELISA kit (MBS730762) by courtesy of MyBioSource lab. Inc., San Diego, CA**.** The file reports the linearity curves obtained with different concentration of synthetic a-syn oligomers and the low cross-reactivity observed with non oligomeric forms of a-syn.
