# Supplementary material for: Abnormal Salivary Total and Oligomeric Alpha-Synuclein in Parkinson’s Disease
Source: PLoS One. 2016 Mar 24;11(3):e0151156. doi: 10.1371/journal.pone.0151156 (PMC4807094; doi:10.1371/journal.pone.0151156)
Supplement: S1 Text — (PDF) [file pone.0151156.s002.pdf]

Human alpha-Synuclein Oligomer ELISA
Sandwich Elisa

Raw data

A	0.069	0.058	------	------	------	------
B	0.105	0.125	------	------	------	------
C	0.233	0.17	------	------	------	------
D	0.53	0.13	------	------	------	------
E	0.91		------	------	------	------
F	1.457		------	------	------	------
G	2.038		------	------	------	------
H	0.073		------	------	------	------

Elisa Distribution

A	blank	blank	------	------	------	------
B	0	spl1	------	------	------	------
C	100	spl2	------	------	------	------
D	250	spl3	------	------	------	------
E	500	------	------	------	------	------
F	1000	------	------	------	------	------
G	2500	------	------	------	------	------
H	blank	------	------	------	------	------

Data processing

	Con pg/ml	OD
A	0	0.105
B	100	0.233
C	250	0.53
D	500	0.91
E	1000	1.457
F	2500	2.038

Four parameter logistic curve regression
Formula: y = (A - D) / [1 + (x/C)^B] + D

A = 2.47587
B = -1.32296
C = 811.41181
D = 0.10247
r^2 = 0.99983

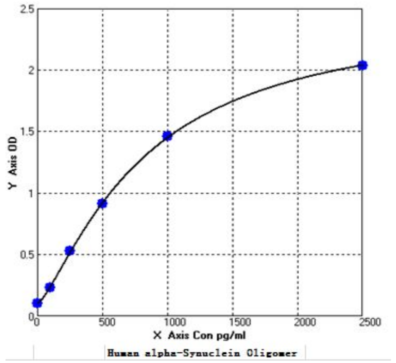

alpha synuclein		con pg/ml	%
1	0.125	0.00967489	0.967489

2	0.17	0.022506257	2.2506257
3	0.13	0.011275324	1.1275324

The antibody is a monoclonal antibody produced by immunizing the mouse with the synthetic peptides. Standards are recombinant protein expressed in E coli. This kit cannot distinguish the different types of alpha synuclein oligomers).

Engineer Menan.
